# Growth, Physiological, and Biochemical Variations in Tomatoes after Infection with Different Density Levels of *Meloidogyne enterolobii*

**DOI:** 10.3390/plants13020293

**Published:** 2024-01-18

**Authors:** Aatika Sikandar, Fangcao Wu, Heliang He, Rana Muhammad Kaleem Ullah, Haiyan Wu

**Affiliations:** State Key Laboratory for Conservation and Utilization of Subtropical Agro-Bioresources, Guangxi Key Laboratory of Agric-Environment and Agric-Products Safety, College of Agriculture, Guangxi University, Nanning 530004, China; aatikasikandar@gxu.edu.cn2217304034@st.gxu.edu.cn (F.W.); 2317304009@st.gxu.edu.cn (H.H.); dr.kaleem@gxu.edu.cn (R.M.K.U.)

**Keywords:** tomato, root-knot nematodes, nematicides, biocontrol, biomass, fermentation

## Abstract

*Meloidogyne enterolobii* is an extremely important plant parasitic nematode. Tomato (*Solanum lycopersicum*) is an essential worldwide vegetable, and *M. enterolobii* poses a major threat to its production. The present research investigated the effects of different levels of inoculum density of *M. enterolobii* (100, 500, 1000, 1500, and 2000 second-stage juveniles (J2s)/plant) on tomato growth, physiological, and biochemical changes at 7, 14, 21, and 28 days post-inoculation (dpi). The negative impact of *M. enterolobii* on plants gradually increased when the inoculum level increased. Therefore, *M. enterolobii* population densities (500–2000 J2s/plant) significantly (*p* < 0.05) reduced plant growth, photosynthetic pigmentation, gas exchange, and chlorophyll fluorescence compared to control plants, while the low population density (100 J2s/plant) showed very little influence. Furthermore, plants with the highest *M. enterolobii* inoculum (2000 J2s/plant) exhibited a greater number of egg masses and galls. The inoculum densities of *M. enterolobii* exhibited a notable correlation with the significant elevation of both malondialdehyde (MDA) and hydrogen peroxide (H_2_O_2_) levels, which are recognized as very detrimental stresses in plants. Similarly, a rise in the activity of several defensive antioxidant enzymes, namely superoxide dismutase (SOD), catalase (CAT), and peroxidase (POD), indicates the defensive mechanism used to combat the oxidative destruction produced by *M. enterolobii*. The specific activity of glutathione (GSH) and ascorbate (ASA) increased as potent antioxidant defense molecules in response to induced oxidative damage. In addition, our findings also demonstrated that the highest population density (2000 J2s/plant) increased the secondary metabolites responsible for scavenging oxidative stress in the plants. However, further research is required to explore the underlying reasons for this phenomenon and to develop efficient chemical or biocontrol strategies for managing *M. enterolobii*.

## 1. Introduction

Root-knot nematodes (RKNs) pose a considerable threat to agricultural crops worldwide and are responsible for substantial yearly output reductions [1]. *Meloidogyne* spp. can be extremely detrimental because of their fast reproduction rate and extensive host diversity [2]. *Meloidogyne enterolobii* is a significant agricultural threat due to its global distribution and ability to infect a broad variety of host plants [3]. According to Collettetal et al. [4], the impacts of *M. enterolobii* might manifest as a reduction in both the quality and quantity of crops. The observable symptoms occurring above ground level include stunted growth, chlorosis, and wilting [5]. Conversely, below ground level, notable symptoms manifest as galls on the root system, which may exhibit significant size and abundance [6]. The yield losses prompted by *M. enterolobii* may exceed 65% [7], making it the most destructive species of RKNs.

Tomato is considered as a highly cultivated vegetable worldwide [8]. Nevertheless, several abiotic and biotic variables contribute to the decreased production of tomatoes [9]. *Meloidogyne enterolobii* is a very virulent species that induces significant root galling in comparison to other species of RKNs [10]. Furthermore, *M. enterolobii* has appeared as a global threat to tomato production because most commercially available resistant tomato cultivars lack resistance to this nematode [11].

The presence of RKNs not only affects the external appearance of plants but also disrupts essential physiological metabolic functions like photosynthesis [12]. The chlorophyll content is an essential indicator of photosynthesis in plants, as it serves as the major channel by which plants acquire energy for their metabolic processes and development [13]. Furthermore, it should be noted that chlorophyll content is particularly sensitive to biotic stresses [14]. Prior research has shown that infection by RKNs leads to a reduction in the chlorophyll concentration in the host plants [15]. Furthermore, previous studies also reported that nematode infestation significantly reduced the growth parameters [16,17].

Plants, under certain stress conditions, generate reactive oxygen species (ROS) [18]. According to Sumanetal et al. [19], one of the most destructive causes of damage in plants that are subjected to biotic stresses such as pathogens is the damage that is induced by ROS. Similarly, when nematodes infect plant tissue, an oxidative burst occurs, which leads to the formation and generation of ROS consisting of malondialdehyde (MDA), hydrogen peroxide (H_2_O_2_), and others [20]. Infected plants activate numerous antioxidant enzymes and non-enzymes for the neutralization of the adverse effects of ROS [21]. Antioxidant enzymes possess the ability to stabilize or neutralize free radicals prior to their oxidation of cellular components [22]. Antioxidant enzymes include catalase (CAT), superoxide dismutase (SOD), peroxidase (POD), etc. [23]. Catalase (CAT) is a crucial antioxidant enzyme that has a significant impact on plant development and stress tolerance [24]. It also regulates the redox balance in plant cells and effectively scavenges H_2_O_2_ generated during photorespiration and mitochondrial electron transfer [25]. Additionally, it plays a significant role in plant defense and stress responses, as well as delays the aging of plants, and regulates the redox balance in plant cells [26,27]. Superoxide dismutase (SOD) is also an antioxidant enzyme that plays a crucial role in the natural defense mechanisms of plants against harmful free radicals and ROS produced as a result of both abiotic and biotic stress [28]. SODs are prevalent metalloenzymes that serve as a primary defense mechanism against ROS [29]. It is a significant enzymatic element involved in the detoxification process of superoxide radicals produced in biological systems [30]. This is achieved by catalyzing the dismutation of superoxide radicals into H_2_O_2_, and subsequently into H_2_O and O_2_, with the help of catalase and peroxidase enzymes [31]. Moreover, peroxidase (POD) is also a crucial antioxidant enzyme that is responsible for removing H_2_O_2_ under oxidative stress in plants [32].

Antioxidant non-enzymes neutralize free radicals by halting their chain reactions [33]. Antioxidant non-enzymes include glutathione (GSH), ascorbate (ASA), etc. [34]. The strong antioxidant non-enzyme glutathione (GSH) plays an important role in controlling various metabolic processes and safeguards the cell membranes by maintaining lower amounts of α-tocopherol and zeaxanthin [35]. Furthermore, it protects proteins from oxidative damage by safeguarding their thiol groups during stress [36]. Additionally, it acts as a building block for both glutathione peroxidase and glutathione S-transferase enzymes [37]. Glutathione has a role in protecting plants against parasitic cyst nematodes [38,39]. Ascorbate (ASA) is a molecule that acts as an antioxidant and is vital for the detoxification of reactive oxygen species [40]. ASA acts as an antioxidant by directly or indirectly scavenging ROS to prevent oxidative damage and improve the plant’s ability to tolerate stress [41]. It also works as a cofactor to control the production and breakdown of different cellular components, including phytohormones [42], thus exhibiting a significant impact on the integration of stress responses and plant growth [43]. Lastly, it can modulate the functions of numerous signaling pathways [44]. Moreover, ASA is a crucial antioxidant in plants, providing them with protection against nematodes [45].

Secondary metabolites are naturally occurring compounds that have a wide variety of physiological functions in plants and are produced through complex metabolic pathways [46]. Secondary metabolites, such as anthocyanin, phenolic flavonoids, etc., play a protective role against biotic and abiotic stressors [47]. Biotic stress can manifest in conjunction with a wide range of organisms, such as bacteria, nematodes, fungi, and viruses [48]. The higher production of secondary metabolites in plants is one of the responses to such stresses [49]. Jha and Mohamed [50] described that plants respond to stress by initiating several metabolic pathways that produce secondary metabolites to aid in tolerance to the biotic stress. Many secondary metabolites are biosynthesized in the phenylpropanoid pathway as a result of a plant’s responses to RKNs [51]. Previous investigations also reported that many morphological, physiological, and biochemical variations occurred in hosts after infestation with nematodes [52]. Keeping this in mind, the current research was planned to determine the influence of various population densities of *M. enterolobii* on the (i) growth, (ii) physiological, and (iii) biochemical changes in tomatoes. Studies on the host-parasite relationship present fundamental knowledge of diseases and could prove beneficial to establishing strategies for controlling nematodes.

## 2. Results

### 2.1. Growth Parameters

The findings demonstrated that the various levels of inoculum (100, 500, 1000, 1500, and 2000 J2s/plant) of *M. enterolobii* significantly decreased plant growth indices (Table 1). It was found that a lower decline in growth parameters was observed in plants infected with inoculum levels of 100 J2 compared to the control plants. However, a notable reduction in shoot length was recorded in plants infected with 500, 1000, 1500, and 2000 J2s/plant, respectively. The plants inoculated with 2000 J2s displayed the most notable decrease in shoot length at 7, 14, 21, and 28 dpi. The plants that were inoculated with 2000 J2s showed a reduction of 52.83%, 47.70%, 44.02%, and 38.41% at 7, 14, 21, and 28 dpi, respectively, as compared with the control group. As nematode population densities increased, a significant reduction in shoot weight was recorded. Therefore, the plants inoculated with the largest number of juveniles (2000 J2s/plant) possessed a considerably reduced shoot weight compared to non-inoculated or control plants. This reduction was 77.60%, 72.15%, 55.34%, and 47.90% at 7, 14, 21, and 28 dpi, respectively. Similarly, plants infected with 2000 J2s/plant showed the most significant decline (*p* < 0.05) in leaf area at 7, 14, 21, and 28 dpi, with reductions of 34.75%, 32.81%, 28.06%, and 21.72%, respectively, as compared to the control group. In comparison, the leaf area of plants infected with 100 J2s/plant exhibited a minimum reduction (2.60%, 6.84%, 8.99%, and 1.97% at 7, 14, 21, and 28 dpi, respectively) as compared to plants in the control group. The plants inoculated with 2000 J2s exhibited a significant reduction (*p* < 0.05) in root length. Specifically, when compared to the control group, the reductions of 51.21%, 48.59%, 43.62%, and 41.26% were found at 7, 14, 21, and 28 dpi, respectively, while the plants infected with 100 J2s/plant exhibited a minimal reduction in root length compared to the control or non-inoculated plants. Similarly, a notable reduction in root weight was observed in plants infected with 500, 1000, 1500, and 2000 J2s/plant. The plants that were inoculated with 2000 J2s showed a reduction of 58.52%, 48.39%, 54.34%, and 60.63% at 7, 14, 21, and 28 dpi, respectively, as compared with the control group.

### 2.2. Nematode Infection

Table 2 displays the infection of *M. enterolobii* at various inoculum levels, including 100, 500, 1000, 1500, and 2000 J2s/plant. A lower number of juveniles per root system (40.80, 55.20, 63.40, and 80.20 juveniles at 7, 14, 21, and 28 dpi, respectively) were detected in plants that were infected with 100 juveniles per plant. In contrast, the plants that were infected with 2000 J2s demonstrated the highest number of juveniles per system (1576.60) at 28 dpi, followed by 1376.60, 1307.40, and 870.60 at 21, 14, and 7 dpi, respectively. The presence of gall formation was observed in all plant specimens that had been affected by nematode infection. A statistically significant and uniform increase in the number of galls was observed as the inoculum level was increased. The plants infected with 2000 J2/plant formed the most galls (117.40 galls) at 28 dpi, followed by 104.80, 92.20, and 71.60 at 21, 14, and 7 dpi, respectively. The number of egg masses was subsequently enhanced (*p* < 0.05) when the inoculum level of *M. enterolobii* increased. At 21 dpi, the plants inoculated with 2000 J2s/plant exhibited the maximum number of egg masses (50.80), while plants inoculated with 100 J2s/plant exhibited the minimum number of egg masses (11.40). Similarly, at 28 dpi, the maximum number of egg masses (63.6) was recorded in plants that were inoculated with 2000 J2s/plant. Similarly, the plants inoculated with 2000 J2s displayed the maximum nematode population per gram of root weight (704.86) at 28 dpi, followed by 672.97, 680.96, and 674.91 at 21, 14, and 7 dpi, respectively.

### 2.3. Photosynthetic Pigments

**Figure 1:**
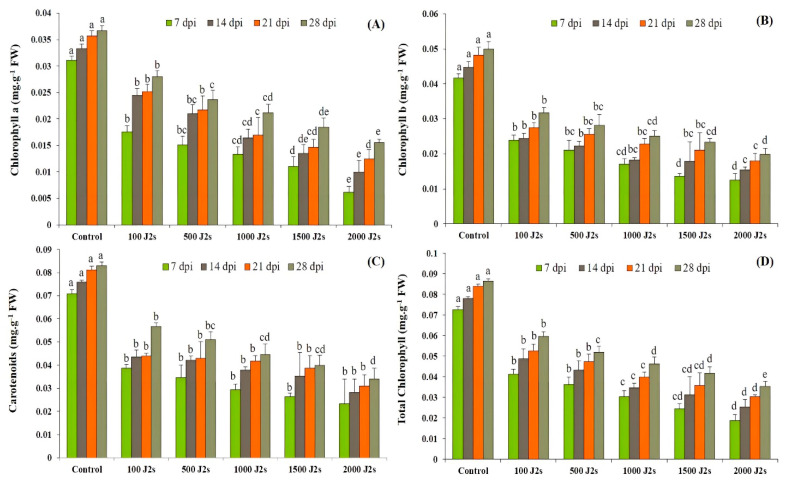
Influence of different inoculum densities of *Meloidogyne enterolobii* on photosynthetic pigments in tomato plants at 7, 14, 21, and 28 dpi. (**A**) Chlorophyll a content; (**B**) Chlorophyll b content; (**C**) Carotenoids content; (**D**) Total chlorophyll content. Bars represent mean ± standard error (*n* = 5). The different letters on the bars represent the significance difference according to Duncan’s multiple range test (*p* < 0.05).

The results showed that photosynthetic pigments decreased significantly (*p* < 0.05) after plants were infected with *M. enterolobii* (100–2000 J2s/plant). At 7, 14, 21, and 28 dpi, the chlorophyll content of plants infected with 2000 J2s was considerably reduced by 80.19%, 70.20%, 65.19%, and 57.58%, respectively, in comparison with the control plants. Similarly, the chlorophyll b contents decreased as the nematodes’ densities increased. The maximum significant reduction was reported in plants infected with 2000 J2s, with reductions of 55.12%, 32.13%, 14.91%, and 48.38%, respectively, in comparison to the control plants at 7, 14, 21, and 28 dpi). The outcomes revealed that the highest significant reductions (67.21%, 62.95%, 61.83, and 58.95%, respectively, at 7, 14, 21, and 28 dpi) in carotenoid contents were observed in plants that were inoculated with 2000 J2s per plant. Moreover, the total chlorophyll content also followed the same trend after being infected with different nematode populations. For instance, the total chlorophyll content in these plants exhibited a maximum decrease of 74.32%, 67.46%, 63.83%, and 59.07% at 7, 14, 21, and 28 dpi, respectively, in comparison to the control plants (Figure 1).

### 2.4. Gas Exchange Parameters

**Figure 2:**
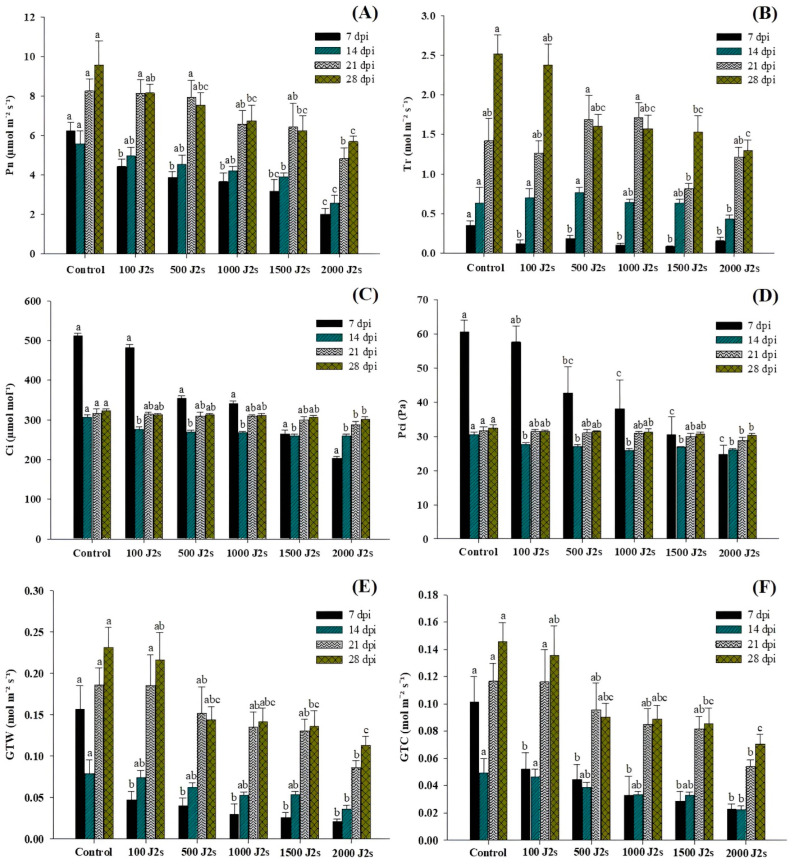
Influence of different inoculum levels of *Meloidogyne enterolobii* on leaf gas exchange measurements in tomato plants at 7, 14, 21, and 28 dpi. (**A**) The net photosynthesis rate (Pn); (**B**) Transpiration rate (Tr); (**C**) Intracellular CO_2_ (Ci); (**D**) Partial pressure of intercellular CO_2_ (PCi); (**E**) Total conductance of H_2_O (GTW); (**F**) Total conductance of CO_2_ (GTC). Bars represent mean ± standard error (*n* = 5). The different letters on the bars represent the significance difference according to Duncan’s multiple range test (*p* < 0.05).

Nematode infection significantly affects the photosynthesis process in host plants. The findings of the study indicated that the presence of nematodes led to a notable reduction in the net photosynthetic rate (Pn), transpiration rate (Tr), intercellular CO_2_ concentration (Ci), partial pressure of intercellular CO_2_ (PCi), total conductance of H_2_O (GTW), and total conductance of CO_2_ (GTC) in all plants that were exposed to an inoculum of 100–2000 J2s in comparison to the control plants (Figure 2). The plants that were treated with 2000 J2s per plant exhibited the maximum decrease in Pn values. At 7, 14, 21, and 28 dpi, the Pn values of the aforementioned plants were considerably reduced by 67.68%, 53.94%, 41.59%, and 40.49%, respectively, in comparison to the control plants (Figure 2A). The data reported in Figure 2B indicate that the transpiration rates (Tr) of tomato plants subjected to a 100–2000 J2s inoculum treatment exhibited a considerable decrease as compared to control plants. The most significant reduction was reported in plants infected with 2000 J2s, with reductions of 55.12%, 32.13%, 14.91%, and 48.38%, respectively, in comparison to control plants at 7, 14, 21, and 28 dpi. Both Ci and PCi values decrease after inoculation with *M. enterolobii* densities ranging from 100 to 2000 J2s/plant (Figure 2C,D). For instance, at 7 dpi, 2000 J2-inoculated tomatoes showed a maximum reduction in Ci and PCi values of 60.36 and 58.97%, respectively, compared to non-inoculated or control plants. The Ci values in plants that were inoculated with 2000 J2s at 14, 21, and 28 dpi exhibited a reduction of 15.11%, 9.20%, and 6.42%, respectively, in comparison with control plants. Moreover, the PCi values of plants infected with 2000 J2s exhibited a reduction of 15.03%, 9.09%, and 6.39% at 14, 21, and 28 dpi, respectively, in contrast to the control group. These foremost outcomes demonstrated that after 7 dpi, there is a drastic decline in the reduction of Ci and PCi values compared to control plants. The data presented in Figure 2E indicate that the GTW values of plants that were treated with 2000 J2s per plant decreased the most significantly. At 7, 14, 21, and 28 dpi, the GTC values in 2000 J2s-inoculated plants showed a remarkable reduction to 86.948%, 54.63%, 53.61%, and 51.35%, respectively, in comparison with the control plants. Similarly, at 7, 14, 21, and 28 days post-inoculation (dpi), plants infected with 2000 J2s showed the most significant reduction (*p* < 0.05), with decreases of 77.51%, 54.69%, 53.73%, and 51.54%, respectively, in comparison with the control group (Figure 2F).

### 2.5. Chlorophyll Fluorescence Traits

**Figure 3:**
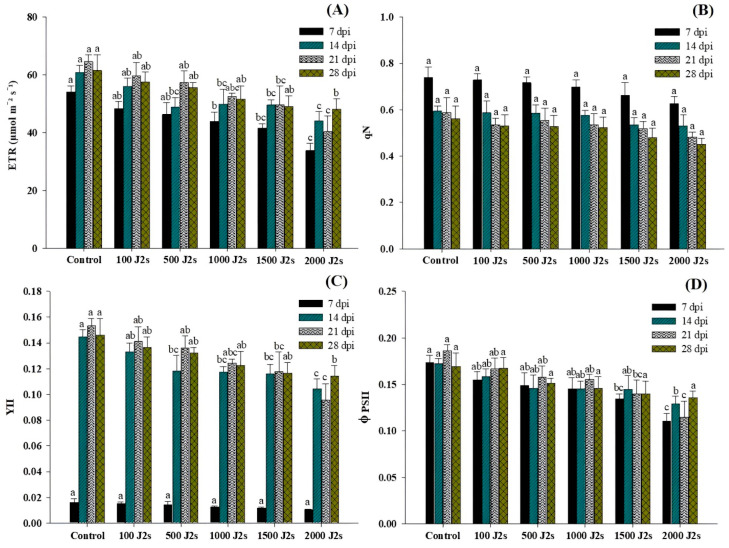
Influence of different inoculum levels of *Meloidogyne enterolobii* on chlorophyll fluorescence measurements in tomato plants at 7, 14, 21, and 28 dpi. (**A**) The electron transport rate of PSII (ETR); (**B**) non-photochemical quenching coefficient (qN); (**C**) PSII quantum yield of light (YII); (**D**) PSII efficiency (φPSII). Bars are mean ± standard error (*n* = 5). Bars represent mean ± standard error (*n* = 5). The different letters on the bars represent the significance difference according to Duncan’s multiple range test (*p* < 0.05).

Figure 3 shows the variation in chlorophyll fluorescence traits in the performance of each group at different time intervals. The different population densities of *M. enterolobii* (100–2000 J2s/plant) exhibited a significant effect on the light energy absorption of tomato leaves. The electron transport rate (ETR), PSII quantum yield of light (YII), and PSII efficacy (φPSII) showed a significant downward trend, while non-photochemical quenching (qN) exhibited a non-significant trend in comparison with control plants. The plants that were treated with 2000 J2s per plant exhibited the maximum decrease in ETR values. At 7, 14, 21, and 28 dpi, the ETR values in 2000J2s inoculated plants showed a remarkable reduction to 37.47%, 27.59%, 37.60%, and 21.81%, respectively, in comparison with the control. In contrast, qN values displayed no significant differences (*p* > 0.05) between all nematode-inoculated plants in comparison with the control plants at different time intervals. The YII values decrease after being inoculated with *M. enterolobii* densities ranging from 100 to 2000 J2s/plant. However, at 7 dpi, YII showed non-significant differences between all nematode-inoculated plants in comparison with the control plants. At 14, 21, and 28 dpi, YII values exhibited a significant difference in comparison with the control plants. The maximum reduction of φPSII values was observed in the plants that were inoculated with 2000 J2s per plant. At 7, 14, 21, and 28 dpi, the PSII efficacy values of the aforementioned plants were considerably reduced by 36.27%, 33.36%, 38.19%, and 20.06%, respectively, in comparison with the control plants.

### 2.6. Antioxidant Enzymes Activities

**Figure 4:**
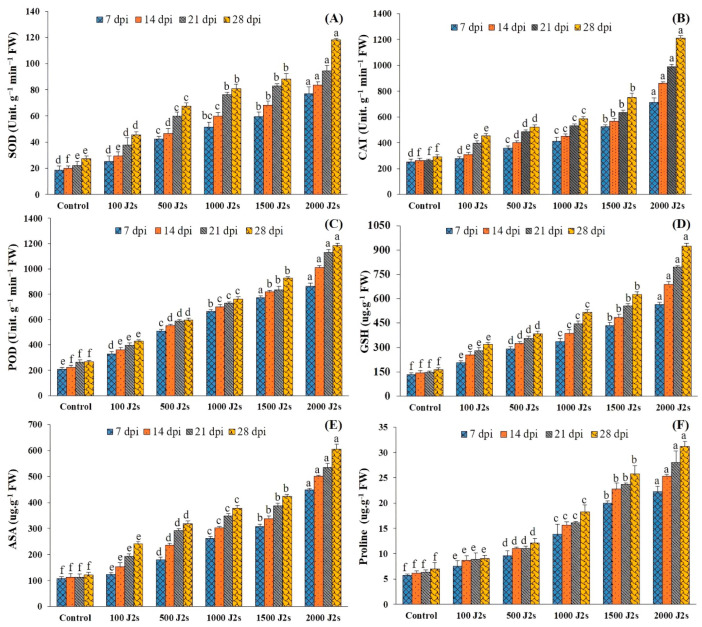
Influence of different inoculum levels of *Meloidogyne enterolobii* on antioxidants and proline content at 7, 14, 21, and 28 dpi. (**A**) Superoxide Dismutase (SOD); (**B**) Catalase (CAT); (**C**) Peroxidase (POD); (**D**) Glutathione (GSH); (**E**) Ascorbate (ASA); (**F**) Proline. Bars represent mean ± standard error (*n* = 5). The different letters on the bars represent the significance difference according to Duncan’s multiple range test (*p* < 0.05).

It was recorded that the activity of all antioxidant enzymes increased significantly in the plants inoculated with various population densities of *M. enterolobii.* The significant maximum increases (*p* < 0.05) of all these antioxidant enzymes were recorded in 2000 J2s-inoculated plants as compared to the control plants. For instance, at 7, 14, 21, and 28 dpi, the SOD activity of the aforementioned plants was enhanced by 4.17, 4.19, 4.25, and 4.34 fold, respectively, in comparison with the control plants (Figure 4A). Similarly, the maximum POD activity (2.82, 3.28, 3.79, and 4.17 fold, respectively, at 7, 14, 21, and 28 dpi) was recorded in 2000 J2s-inoculated plants (Figure 4B). Moreover, the SOD activity also followed the same trend after being infected with the different nematode populations. The unit SOD activity of plants inoculated with 2000 J2s/plant exhibited a maximum increase of 44.12, 4.58, 4.32, and 4.45 fold at 7, 14, 21, and 28 dpi, respectively, compared with the control (Figure 4C).

### 2.7. Non-Enzymatic Antioxidants

The specific activity of GSH increased significantly in plants inoculated with different numbers of *M. enterolobii* (100–2000 J2s per plant). At 7, 14, 21, and 28 dpi, the GSH values of 2000 J2s inoculated plants increased significantly by 4.28 fold, 4.79 fold, 5.38 fold, and 5.58 fold relative to the control, respectively (Figure 4D). In a similar way, the total ascorbic acid (ASA) content of tomato plants inoculated with 100–2000 J2s inoculum showed a significant increase. At 7, 14, 21, and 28 dpi, plants infected with 2000 J2s exhibited a significant increase (*p* < 0.05) in ASA content, with increases of 4.19 fold, 4.46 fold, 4.75 fold, and 4.96 fold, respectively, as compared to the control plants (Figure 4E). However, plants inoculated with 100 J2s had the minimum increase in all antioxidant non-enzymes compared to the control plants.

### 2.8. Proline Content (Osmolyte)

Figure 4F demonstrates that proline content was highest in plants with various nematode populations’ densities in comparison with the control plants. As the inoculum density increased, the proline content also increased. The highest increases in proline content (3.91, 4.14, 4.44, and 4.45 fold) were recorded in 2000 J2s-inoculated plants at 7, 14, 21, and 28 dpi, respectively. Comparatively, the proline content of 100 J2s-infected plants increased minimally (1.34, 1.41, 1.39, and 1.29 fold at 7, 14, 21, and 28 dpi, respectively) compared to control plants.

### 2.9. Stress Indices

**Figure 5:**
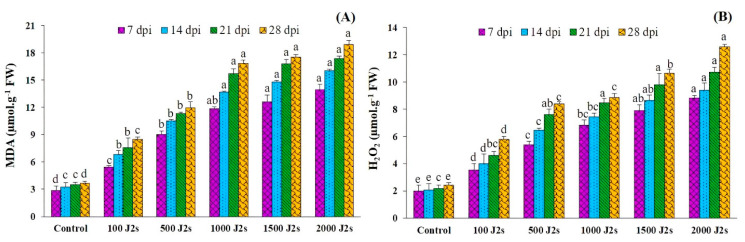
Influence of different inoculum levels of *Meloidogyne enterolobii* on oxidative stress responses at 7, 14, 21, 28 dpi. (**A**) Lipid peroxidation (malondialdehyde (MDA)); (**B**) Hydrogen peroxide (H_2_O_2_). Bars represent mean ± standard error (*n* = 5). The different letters on the bars represent the significance difference according to Duncan’s multiple range test (*p* < 0.05).

The oxidative burst was triggered in *M. enterolobii*-infected tomato plants (Figure 5). The plants that were infected with 2000 J2s exhibited the highest recorded levels of H_2_O_2_, in contrast to the control plants. The content of H_2_O_2_ in 2000 J2s-inoculated plants significantly increased (*p* < 0.05) by 4.42, 4.53, 4.91, and 5.19 times compared to the control plants at 7, 14, 21, and 28 dpi (Figure 5A). The quantification of malondialdehyde (MDA) levels in tomato plants serves as an indicator of the occurrence of lipid peroxidation. The results presented in Figure 5B indicate a substantial increase (*p* < 0.05) in the malondialdehyde (MDA) content in tomato plants inoculated with *M. enterolobii* in comparison with the control or non-inoculated plants. The maximum increases in MDA content (4.88, 4.95, 4.98, and 5.18 fold) were recorded in 2000 J2s-inoculated plants at 7, 14, 21, and 28 dpi, respectively.

### 2.10. Secondary Metabolites

**Figure 6:**
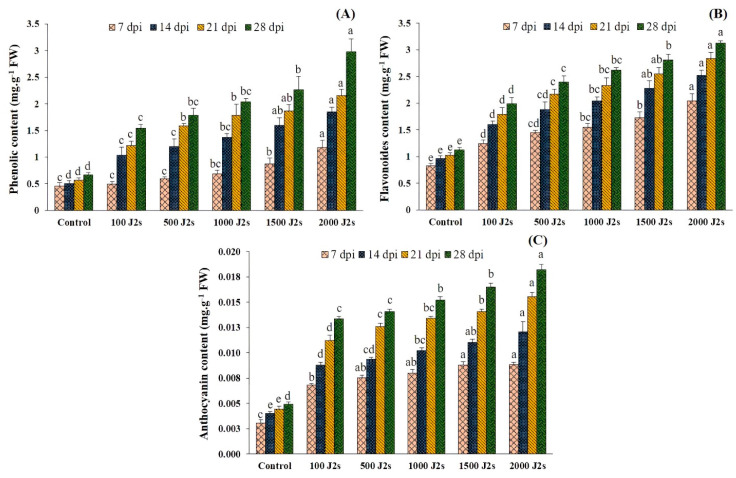
Influence of different inoculum levels of *Meloidogyne enterolobii* on secondary metabolites at 7, 14, 21, 28 dpi. (**A**) Total phenolic content; (**B**) Total flavonoid content; (**C**) Total anthocyanin content. Bars represent mean ± standard error (*n* = 5). The different letters on the bars represent the significance difference according to Duncan’s multiple range test (*p* < 0.05).

The total phenolic, flavonoid, and anthocyanin contents exhibited an upward trend in the plants that were infected with nematodes as compared to the control group (Figure 6). Moreover, the total phenol content of tomato plants inoculated with 100–2000 J2s showed a significant increase. At 28 dpi, the highest observed increase was 4.48 fold in plants that were inoculated with 2000 J2s, compared to the control. This was followed by a rise of 3.81 fold at 21 dpi, 3.69 fold at 14 dpi, and 2.60 fold at 7 dpi. Similarly, the outcomes revealed significant increases in total flavonoid content in plants infected with 2000 J2s/plant at various time points. The maximum fold increases were recorded at 7, 14, 21, and 28 dpi, with values of 2.47, 2.63, 2.76, and 2.78, respectively. The anthocyanin content exhibited the same trend as total phenolic and flavonoid contents. The anthocyanin content exhibited a substantial rise (*p* < 0.05) in response to the progressive increase in nematode population densities observed throughout the study. Similarly, the highest observed increase in total anthocyanin content was 3.70 fold in plants infected with 2000 J2s at 28 dpi. Following this, there was a rise of 3.52 at 21 dpi, 2.99 at 14 dpi, and 2.90 fold at 7 dpi when compared with the control plants.

## 3. Discussion

Root-knot nematodes produce the formation of enlarged cells inside the roots, causing many alterations in host plants [53]. Despite the successful invasion of second-stage juveniles (J2s) into the roots and subsequent initiation of gall development, the overall effect on plant growth remained negligible [54]. *Meloidogyne enterolobii* elicited a range of morphological and physiological alterations [4]. The investigation of the biochemistry underlying the interaction between hosts and pathogens is of utmost importance in the characterization of plant diseases. Thus, the current research was planned to explore the influences of different population densities of *M. enterolobii* on growth, physiological, and biochemical changes in tomatoes.

It was observed that the growth of tomato plants exhibited a noticeable reduction as the inoculum level of *M. enterolobii* (ranging from 100 to 2000 J2s) increased. The findings of this research showed that the highest inoculum level (2000 J2s/plant) resulted in a substantial decrease in many parameters. Specifically, there was a decrease of 52.83% in shoot length, 77.60% in shoot weight, 34.75% in leaf area, 51.21% in root length, and 60.63% in root weight as compared to the control plants. Our findings agree with Danish et al. [55] that when inoculum levels of RKNs increased (from 100 to 4000 J2s/plant), *Trachyspermum ammi* (L.) plant development was drastically reduced. The current research confirms previous findings that *M. enterolobii* infection causes a reduction in plant biomass via the formation of root galls. The previous researchers believed that decreased growth characteristics resulted from RKNs’ obstruction of the vessels involved in water and nutrient transmission [56].

The results of the present study indicate a statistically significant positive correlation between the degree of inoculum and the number of juveniles, egg masses, and galls of nematodes. For instance, the plants that were subjected to the highest inoculum level (2000 J2s/plant) exhibited the greatest number of egg masses and galls. In contrast, when the initial inoculum level was 100 J2, there were considerably fewer galls, egg masses, and juveniles present in the root system. Previous research findings also demonstrated that the pathogenicity of RKNs was found to be in association with the degree of inoculum and the number of egg masses and galls in greenhouse conditions. Specifically, it was revealed that the number of egg masses and galls increased significantly when the inoculum levels reached or exceeded 1000 J2s in capsicum plants [57]. In another investigation, it was further demonstrated that the quantity of egg masses, galls, and population of nematodes per root system exhibits a gradual increase as the inoculum (ranging from 100 to 4000 J2s per plant) increases [55].

The findings derived from the current investigation additionally revealed a significant reduction in chlorophyll contents in tomato plants infected with *M. enterolobii*. In a previous study, the nematode infections in tomato plants reduced or altered photosynthetic pigments in tomato plants [58]. Chen et al. [59] also determined that the leaf chlorophyll content of rice plants decreased after infection with *M. graminicola*, with an inoculum density ranging from 500 to 2000 J2s per plant. Further demonstrated that the tomato plant inoculation with the highest inoculum (2000 J2s/plant) resulted in a considerable reduction in chlorophyll a (80.19%), chlorophyll b (55.12%), carotenoids (67.21%), and total chlorophyll content (74.32%) as compared to the control or non-inoculated plants.

Nematode infection poses a biotic stress factor for plants, resulting in detrimental effects on roots and disrupting the transportation of minerals and water [60]. Consequently, this interference impacts the process of photosynthesis [61]. The outcomes of the current investigation demonstrate that the inoculation of nematodes resulted in a noteworthy decrease in various leaf gas exchange characteristics. The previous research investigation was conducted with rice plant inoculation with RKN juveniles (100, 500, 1000, and 2000 J2s/plant), which resulted in a significant decrease in the photosynthetic process [59]. Therefore, the density of the nematode population was adversely associated with the photosynthesis rate.

The present research revealed that *M. enterolobii* substantially decreased the chlorophyll fluorescence parameters, including electron transport rate (ETR), PSII quantum yield of light (YII), and PSII effectiveness (φPSII). The current study allowed to observe a significant decrease in chlorophyll fluorescence parameters as a result of increased nematode population levels (100–2000 J2s/plant). In this study, we observed that the chlorophyll fluorescence drastically decreased as the nematode population increased. The activity of chlorophyll in the photosystem reaction center is directly correlated with the level of chlorophyll fluorescence. Our findings are consistent with those of Ghasemzadeh et al. [62], indicating that the chlorophyll fluorescence characteristics were significantly influenced by increasing the nematode inoculum. In a broader context, the plants subjected to nematode-induced stress exhibited a decline in the efficiency rate of photosystem II in both light and dark circumstances as nematode levels increased.

Nematode-infected plants exhibited maximum levels of both enzymatic antioxidants (SOD, CAT, and POD) and non-enzymatic antioxidants (GSH and ASA) as compared to the control plant. Khajuria and Ohri [63] also observed that nematode infection of tomato plants increased the activity of SOD, CAT, POD, GPOX, etc. During the present study, the antioxidant enzymes and non-enzymatic antioxidants of tomato plants increased following infection with *M. enterolobii* at an inoculum density ranging from 500 to 2000 J2s per plant. Significant maximum increases (*p* < 0.05) were observed in the plants infected with 2000 J2s in comparison with the control plants. The prior studies additionally revealed that the antioxidant enzymes and non-enzymes exhibited an increase in response to higher levels of nematode inoculation [55,64]. Moreover, proline serves as an osmolyte that protects plants under stressful conditions [65]. In this research, proline levels were shown to appear significantly higher in nematode-infected plants. Consistent with the research conducted by Tikoria et al. [58] and Khajuria and Ohri [63], we also recorded that the proline level increased considerably after the nematode invasion of the tomato plants.

Our study also showed that *M. enterolobii* stress increases oxidative damage in tomato plants, as measured by increased levels of oxidative stress markers such as MDA and H_2_O_2_. Khanna et al. [66] reported a similarly dramatic increase in oxidative stress markers in tomato plants inoculated with nematodes. Furthermore, we found that oxidative stress increased rapidly along with *M. enterolobii* inoculum (100–2000 J2s). These findings align with the research conducted by Afifi et al. [64], who observed an increase in both malondialdehyde (MDA) and hydrogen peroxide (H_2_O_2_) levels in several hosts in response to varied levels of inoculation with *M. incognita*, *Rotylenchulus reniformis*, and *Tylenchulus semipenetrans*. While the oxidants are increased at different rates depending on the nematode type, inoculum, and host plant.

Secondary metabolites are produced by plants as a consequence of nematode infection [67]. Previous studies demonstrated that these metabolites possess the ability to increase the plant’s defense system and antioxidant properties after being subjected to root-knot nematodes (RKNs) [52,58]. In the present work, it was observed that there was a noteworthy enhancement in the levels of total phenolic, flavonoid, and anthocyanin contents due to an increase in nematode population levels (ranging from 100 to 2000 J2s per plant). This aligns with the findings of Tsaniklidis et al. [68], who reported that the levels of total phenols and flavonoids increased when three inoculation regimes of *M. javanica* juveniles were applied (0, 100, and 850 J2s/plant), in comparison to the control group. Khanna et al. [66] also documented an elevation in concentrations of phenolic chemicals, after infection with RKNs.

## 4. Materials and Methods

### 4.1. Plant Material and Nematodes Inoculum

The greenhouse experiment was conducted at the nematology laboratory of Guangxi University in China. The Zhongza 09 tomato seeds were disinfected with 75% ethanol and 2.5% sodium hypochlorite for 1 min. Subsequently, 95% ethanol was added to the seeds for 2–3 min. Then seeds were properly rinsed with sterilized water [69]. The sterilized seeds were planted in plastic pots, with one seed per pot. Then the plants were irrigated with tap water as per requirements in the greenhouse, with a temperature range of 26–32 °C, a light exposure of 14 h, followed by a dark period of 10 h, and a relative humidity of 85%.

*Meloidogyne enterolobii* used in this study was initially collected from *Acalypha australis* plants from a greenhouse (22°50′ N; 108°17′ E) located in Guangxi Province, China [6]. The identification of the nematode species was accomplished by analyzing perineal patterns and esterase behaviors. The sequence characterized amplified region–polymerase chain reaction (SCAR-PCR) was used for molecular identification. A single egg mass was obtained from an identified population reared on nematode-susceptible tomatoes in a greenhouse. To provide an adequate quantity of nematodes for the research, tomato plants were infected with nematodes and cultivated in a greenhouse for 90 days. Then, the infected plants were cut at the soil level, and the roots were washed under running tap water to remove all soil and debris. The mature egg masses were picked from the roots of infested plants. The 0.05% sodium hypochlorite was poured and shaken for 1–2 min. The egg mixture was transferred in a 500-sieve mesh and carefully rinsed through sterilized water. By using the Baermann funnel technique [70], second-stage juveniles (J2s) were allowed to hatch in sterile water at 85% humidity and 28 °C.

### 4.2. Treatment Plan

Approximately 100, 500, 1000, 1500, and 2000 J2s/plant were used to inoculate the plants, while the control plants received sterile water inoculation. For measuring the desired parameters, the five randomly selected plants of each treatment and control group were examined at four-time intervals (7, 14, 21, and 28 dpi).

### 4.3. Assessment of Growth Parameter and Determination of Nematode Infection

A random selection of five plants was made from each treatment at 7, 14, 21, and 28 dpi in order to assess the variability in growth metrics. The shoot length (cm), leaf area (cm^2^), shoot weight (g), root length (cm), and root weight (g) were assessed as growth parameters. For the determination of nematode infection, the tomato roots were properly cleaned with tap water, and the number of egg masses and galls was carefully assessed. Then, it was placed into a 0.5% NaOCl solution and left for 2–3 min. After that, the roots were rinsed under running tap water. After that, the roots were heated for 2 min at 80 °C in an acid fuchsin solution (0.35 g acid fuchsin, 25 mL acetic acid, and 75 mL distilled water). After cooling, the roots were removed from the solution and rinsed with tap water [71]. Then, roots were treated with acidified glycerin and examined carefully under a microscope (OLYMPUS SZX2-ILLT, Tokyo, Japan) to identify the RKN stages.

### 4.4. Total Chlorophyll Contents

The chlorophyll a, b, and carotenoids were measured according to [58]. About 0.2 g of fresh leaf was cut and transferred into 5 mL of 80% acetone. Then kept in a light-restricted environment for a duration of 24 h at 0–4 °C. After that, the resultant solution was centrifuged at 8000 rpm for 5 min. The absorbance of the supernatant was read at 470 nm, 646 nm, and 663 nm by using a spectrophotometer (Multiscan GO 1510, Thermo Fisher Scientific Oy, Ratastie, Finland).

### 4.5. Leaf Gas Exchange and Chlorophyll Fluorescence Parameters Characteristics

The gas exchange characteristics, particularly Transpiration rate (Tr), Photosynthesis rate (Pn), intercellular CO_2_ concentration (Ci), partial pressure of intercellular CO_2_ (PCi), the total conductance of water (GTW), and Total conductance of CO_2_ (GTC) were estimated by the portable infrared gas analyzer photosynthetic system LI-6800XT (GFS-3000; Heinz Walz, Effeltrich, Germany) between 08:30 and 11:00. The readings were taken from three consecutive fully expanded leaves per plant from at least five different places. The parameters were 28 ± 0.8 °C temperature, 1000 ± 14 µmol m^−2^ s^−1^ light intensity, 0.5 µmol s^−1^ flow rate, and 350 ± 3 µmol CO_2_ mol^−1^ CO_2_ concentration. The measurement of chlorophyll fluorescence was estimated by a portable saturation pulse fluorimeter (PAM-2100, 2030-B; Walz, Effeltrich, Germany) between 07:00 and 08:00. The maximum fluorescence yield (Fm) was measured with a 0.8 s saturating pulse at >8000 mmol m^−2^ s^−1^, while the minimum fluorescence yield (Fo) of dark-adapted leaves was determined by measuring light at 0.5 mmol m^−2^ s^−1^ at a frequency of 0.6 kHz. After this, the potential quantum yield of PSII (Fv/Fm) was calculated, where Fv is the maximum variable fluorescence (Fv = Fm − Fo). Prior to the measurement of non-photochemical quenching (qN), leaves were light-adapted for about 12 min. The qN was obtained at 1500 mol m^−2^ s^−1^ PAR. Moreover, the actual PSII efficiency (φPSII) was calculated as φPSII = Fv/Fm × qP.

### 4.6. Measurement of Antioxidant Enzymes

All enzyme extraction processes were conducted at 4 °C. About 2 mL of an extraction solution (0.1 M potassium phosphate buffer with 0.5 mM EDTA, pH 7.0) was used to macerate a 0.5 g sample of roots, and the mixture was centrifuged for 15 min at 15,000× *g* at 4 °C. The resultant extract was used for the determination of the activities of superoxide dismutase (SOD), catalase (CAT), and peroxidase (POD) by following these methods.

The method proposed by Yong et al. [72] was used to evaluate the activity of superoxide dismutase (SOD) by observing the photoreduction of nitroblue tetrazolium (NBT). The 3 mL reaction mixture contained 50 mM phosphate buffer (pH 7.8), 50 mM sodium carbonate, 75 µM NBT, 13 mM methionine, 1.0 mM EDTA, 10 µM riboflavin, and 100 µL enzyme extract were added into plastic cuvettes. A control reaction was achieved without the enzyme extract. The cuvettes were then left in the light for 5 min before their absorbance at 560 nm was measured using a spectrophotometer. Subsequently, the cuvettes were subjected to illumination for a duration of 5 min. The process of photoreduction of NBT results in the formation of a blue formazan. After that, the absorbance was measured at a wavelength of 550 nm using a spectrophotometer. The SOD activity was expressed as Unit/g min FW.

The catalase (CAT) activity was determined by mixing 100 µL of enzyme extract with 1.9 mL of phosphate buffer (50 mM, pH 7.0) and 1 mL of 3% H_2_O_2_. To quantify CAT enzyme activity, the absorbance at 240 nm was monitored for changes in response to H_2_O_2_ consumption every 15 s for three minutes [73]. The CAT activity was measured in Unit/g min FW.

The determination of peroxidase (POD) activity was conducted by assessing the peroxidation of H_2_O_2_ using guaiacol as an electron donor. The 3 mL reaction mixture contained 50 mM phosphate buffer (pH 7.0), 40 mM hydrogen peroxide, 20 mM *guaiacol* solution, and 100 µL enzyme extract. At a wavelength of 470 nm, we measured the increase in absorbance that resulted from the oxidation of guaiacol every 15 s for three minutes [74]. The POD activity is expressed as Unit/g min FW.

### 4.7. Measurement of Non-Enzymatic Antioxidants

A total of 0.2 g of fresh roots were subjected to homogenization using 5 mL of 0.1% solution of trichloroacetic acid (TCA), and the mixture was centrifuged for 15 min at 15,000× *g* at 4 °C. The resultant supernatant was used for the determination of total glutathione (GSH) and total ascorbate (ASA) by the following methods.

The total GSH was determined by mixing 0.5 mL of the supernatant was combined with 1 mL of 200 mM PBS (pH 7.0), 0.15 mL of 0.5 mM NADPH (with 7 mM EDTA), and 0.15 mM of dithiobis-2-nitrobenzoic acid (DTNB). Five units of glutathione reductase (GR) were used to initiate the reaction, which was then incubated at 27 °C for 30 min. The absorbance of the reaction mixture was measured at 412 nm. On the basis of a standard curve generated with known GSH concentrations, the total GSH content was determined [75]. The total GSH was expressed as µg g^−1^ FW.

A reaction mixture comprising 0.2 mL of the supernatant, 0.2 mL of deionized water, and 0.5 mL of 150 mM phosphate buffer (pH 7.4, containing 5 mM EDTA) was used to measure the total ascorbate (ASA). After that, 0.4 mL of 44% (*v*/*v*) phosphoric acid, 0.4 mL of 10% TCA (*w*/*v*), 0.2 mL of 3% FeCl3 (*w*/*v*), and 0.4 mL of α- α dipyridyl in 70% ethanol (*v*/*v*) were added to the reaction mixture, and as a result, color developed in it. Then the reaction mixtures were subjected to incubation for 40 min at 40 °C, after which the absorbance was measured at a wavelength of 532 nm [76]. The amount of ascorbic acid (ASA) was measured in µg g^−1^ FW using a standard curve created from ASA.

### 4.8. Proline Contents

The determination of proline content included the grinding of 0.25 g of root in 5 mL of 3% sulfosalicylic acid, and the mixture was centrifuged at a speed of 8000 rpm at 25 °C for 5 min. Equal amounts of plant extract, ninhydrin solution, deionized water, and glacial acetic acid were added. Then the mixture was heated to 100 °C for 1 h inside a boiling water bath. Subsequently, immediately cool the mixture to stop the reaction before adding toluene. The mixture was vigorously stirred using a vortex shaker for 10–15 s, after which it was allowed to settle without any further disturbance. The absorbance of the uppermost layer was measured at a wavelength of 520 nm using a spectrophotometer [77].

### 4.9. Stress Indices

A homogenate was prepared by macerating 0.25 g of fresh roots in 5 mL of 0.1% (*w*/*v*) solution of trichloroacetic acid (TCA), followed by centrifugation at 4 °C and 10,000 rpm for 10 min. The supernatant obtained from the procedure was then used for the subsequent analysis of hydrogen peroxide (H_2_O_2_) and malondialdehyde (MDA) contents.

In order to measure the content of hydrogen peroxide (H_2_O_2_), 0.1 mL of the supernatant was mixed with 1 mL of 50 mM potassium phosphate buffer and 1 mL of 1 M potassium iodide. The spectrophotometric (Multiscan GO 1510, Thermo Fisher Scientific Oy, Ratastie, Finland) measurement of the reaction mixture’s absorbance was then performed at a wavelength of 390 nm. The content of H_2_O_2_ was calculated based on a standard curve created with known H_2_O_2_ concentrations [78]. The outcomes were expressed as µmol g^−1^ FW.

Lipid peroxidation (malondialdehyde (MDA)) was measured as described by Yang et al. [79]. About 2 mL of the supernatant was mixed with 2 mL of 0.67% thiobarbituric acid (TBA), heated at 100 °C for 20 min, and then cooled to room temperature. Absorbance at 450 nm, 532 nm, and 600 nm was measured for each aliquot. The MDA contents were expressed as µmol g^−1^ FW.

### 4.10. Secondary Metabolites

About 0.2 g of root was homogenized in cold 70% (*v*/*v*) methanol containing 28% (*v*/*v*) ethanol and 2% (*v*/*v*) formic acid. The homogenate was extracted using an ultrasonic shaker at 300 rpm for 2 h at 30 °C. After that, the homogenate was centrifuged at 10,000 rpm for 10 min at 4 °C. The supernatant was filtered through a 0.45 μm filter membrane. After that, the resultant root extract was stored at 4 °C for the subsequent analysis of flavonoid, phenolic, and anthocyanin contents.

The total flavonoid content was estimated with the help of the Aluminum chloride colorimetric technique by aluminum trichloride (AlCl_3_) and sodium hydroxide (NaOH). About 2 mL root extract and 6 mL methanol were added into 10 mL distilled water. After that, 0.4 mL 10% AlCl3 and 0.2 mL 1M CH_3_COOK were mixed in a mixture. The subsequent solution was incubated for 30 min in a dark place at room temperature. The absorbance was measured at a wavelength of 420 nm. Quercetin (1 mg/mL) was used to make a calibration graph. The outcomes were calculated to be quercetin’s equivalent (QE) mg/g extract [80].

Total phenol content was estimated by the Folin–Ciocalteu phenol reagent technique derived from the methodology described by Sikandar et al. [81]. About 2 mL extract and 4 mL of 2% Na_2_CO_3_ were added into 5 mL 10% Folin–Ciocalteu’s reagent (C_10_H_5_NaO_5_S) and incubated at room temperature for 15 min in a dark area. The absorbance was measured at a wavelength of 765 nm. Gallic acid was used to generate the calibration graph using a sequence of concentrations. The outcomes were calculated to be Gallic acid equivalent (GAE) mg/g extract.

The pH differential was employed as a means to measure the anthocyanin content. The root extract followed dilution to achieve pH levels of 1.0 and 4.5 using a buffer solution. Subsequently, the absorbance values at wavelengths of 510 nm and 700 nm were determined for each pH level. The difference between them was used to compute the total anthocyanin content [82].

### 4.11. Statistical Analysis

The recorded data were organized using the MS Excel 2016 program and analyzed by statistical analysis using the IBM-SPSS Statistics 25.0 version software. The data were subjected to statistical analysis using analysis of variance (ANOVA), followed by the Duncan multiple range test with a significance level of *p* < 0.05. Sigma Plot 10.0 and MS Excel were used to create bar graphs.

## 5. Conclusions

Based on the results of the present study, we conclude that the presence of *M. enterolobii* infection leads to notable changes in the morphological, physiological, and biochemical characteristics of tomato plants. The results showed that increasing the amount of inoculum resulted in a substantial reduction (*p* < 0.05) in growth, photosynthetic pigments, gas exchange, and chlorophyll fluorescence parameters in plants. There was a clear association observed between the population densities of *M. enterolobii* and the elevated levels of MDA and H_2_O_2_, which are well-known to cause considerable stress in plants. In addition, the levels of antioxidant enzymes (SOD, CAT, and POD) and non-enzymatic antioxidants (GSH and ASA) exhibited an increase in response to higher inoculum levels. The findings of the current research provide unequivocal evidence that *M. enterolobii* has a considerable impact on the growth, physiology, and biochemical characteristics of tomatoes. The tomato plants were severely damaged due to *M. enterolobii*. Therefore, it is essential to establish control strategies to ensure profitable tomato production in areas where higher nematode populations are present. This study on the host-parasite relationship presents fundamental knowledge of infection caused by different population densities of *M. enterolobii* and could provide a fundamental basis for establishing strategies for controlling this nematode. Therefore, it is essential to conduct further research in order to explore the underlying reasons for this phenomenon and create effective techniques for controlling *M. enterolobii*.

## Figures and Tables

**Table 1:** Influence of *Meloidogyne enterolobii* on growth parameters of tomato plants.

Days Post-Inoculation (dpi)	Level of Inoculum (Number of J2s)	Growth Parameters				
		Shoot Length (cm)	Shoot Weight (g)	Leaf Area (cm^2^)	Root Length (cm)	Root Weight (g)
7	Control	15.71 ± 1.96 ^a^	14.11 ± 1.33 ^a^	4.23 ± 0.69 ^a^	9.92 ± 0.97 ^a^	3.11 ± 0.17 ^a^
	100	11.97 ± 1.26 ^b^	12.15 ± 1.10 ^b^	4.12 ± 0.76 ^a^	8.74 ± 0.59 ^ab^	2.98 ± 0.96 ^a^
	500	10.93 ± 1.16 ^bc^	10.57 ± 1.57 ^b^	3.72 ± 0.95 ^ab^	7.54 ± 1.08 ^bc^	2.35 ± 0.08 ^b^
	1000	9.49 ± 2.58 ^bcd^	8.54 ± 1.25 ^c^	3.63 ± 0.59 ^ab^	7.02 ± 1.86 ^bc^	2.03 ± 0.03 ^bc^
	1500	8.698 ± 2.37 ^cd^	6.06 ± 1.37 ^d^	3.59 ± 0.64 ^ab^	6.56 ± 2.43 ^cd^	1.59 ± 0.02 ^cd^
	2000	7.41 ± 1.26 ^d^	3.16 ± 1.41 ^e^	2.76 ± 0.85 ^b^	4.84 ± 1.05 ^d^	1.29 ± 0.01 ^d^
14	Control	18.45 ± 1.64 ^a^	14.22 ± 0.95 ^a^	5.12 ± 0.69 ^a^	11.32 ± 1.08 ^a^	3.72 ± 0.77 ^a^
	100	15.71 ± 2.03 ^b^	12.25 ± 1.35 ^b^	4.77 ± 0.99 ^a^	9.78 ± 1.43 ^ab^	3.58 ± 0.94 ^a^
	500	13.25 ± 2.82 ^bc^	11.26 ± 1.35 ^b^	4.16 ± 0.78 ^ab^	8.90 ± 0.94 ^b^	3.10 ± 0.12 ^a^
	1000	11.01 ± 1.59 ^cd^	8.45 ± 1.66 ^c^	4.15 ± 0.65 ^ab^	8.82 ± 1.37 ^b^	2.39 ± 0.05 ^b^
	1500	10.73 ± 1.71 ^cd^	6.70 ± 0.99 ^d^	4.15 ± 0.61 ^ab^	8.42 ± 1.04 ^b^	2.01 ± 0.03 ^b^
	2000	9.65 ± 1.59 ^d^	3.96 ± 1.40 ^e^	3.44 ± 0.65 ^b^	5.82 ± 1.54 ^c^	1.92 ± 0.01 ^b^
21	Control	21.22 ± 2.03 ^a^	21.27 ± 1.83 ^a^	6.45 ± 0.50 ^a^	12.06 ± 0.95 ^a^	4.49 ± 0.42 ^a^
	100	19.82 ± 1.89 ^a^	19.97 ± 0.62 ^ab^	5.87 ± 0.52 ^b^	10.44 ± 0.76 ^ab^	3.98 ± 0.21 ^b^
	500	18.64 ± 2.63 ^ab^	18.65 ± 1.67 ^bc^	5.83 ± 0.29 ^b^	9.16 ± 1.91 ^b^	3.40 ± 0.17 ^c^
	1000	15.68 ± 2.91 ^bc^	16.59 ± 1.43 ^c^	5.58 ± 0.40 ^bc^	8.54 ± 1.26 ^bc^	2.56 ± 0.06 ^d^
	1500	13.64 ± 2.22 ^cd^	11.88 ± 1.57 ^d^	5.17 ± 0.35 ^cd^	6.96 ± 1.68 ^c^	2.14 ± 0.02 ^e^
	2000	11.88 ± 1.66 ^d^	9.50 ± 1.47 ^d^	4.64 ± 0.52 ^d^	6.80 ± 2.10 ^c^	2.05 ± 0.01 ^e^
28	Control	27.49 ± 1.26 ^a^	22.59 ± 1.55 ^a^	7.09 ± 0.75 ^a^	13.38 ± 1.15 ^a^	5.69 ± 0.22 ^a^
	100	22.15 ± 1.44 ^b^	21.23 ± 1.71 ^ab^	6.95 ± 0.91 ^ab^	11.70 ± 0.83 ^ab^	4.27 ± 0.37 ^b^
	500	21.17 ± 1.98 ^bc^	19.61 ± 1.91 ^b^	6.40 ± 0.55 ^abc^	10.84 ± 1.70 ^bc^	3.58 ± 0.09 ^c^
	1000	19.55 ± 2.08 ^cd^	16.93 ± 1.46 ^c^	6.32 ± 0.42 ^abc^	10.74 ± 2.27 ^bc^	3.06 ± 0.05 ^d^
	1500	18.75 ± 2.07 ^de^	13.62 ± 1.60 ^d^	6.06 ± 0.48 ^bc^	9.16 ± 2.18 ^cd^	2.22 ± 0.02 ^e^
	2000	16.93 ± 1.06 ^e^	11.77 ± 1.14 ^d^	5.55 ± 0.52 ^c^	7.86 ± 1.63 ^d^	2.24 ± 0.01 ^e^

Data represents Mean ± standard deviation (*n* = 5). The different letters inside a column represent the significance difference according to Duncan’s multiple range test (*p* < 0.05).

**Table 2:** Influence of *Meloidogyne enterolobii* on nematodes population per root system.

Days Post-Inoculation (dpi)	Nematodes Population				
	Level of Inoculum (Number of J2s)	Number of Juveniles per Root System	Number of Galls per Root System	Egg Masses per Root System	Nematodes Population per Gram Root Weight
7	Control	0 ± 0 ^f^	0 ± 0 ^f^	-	0 ± 0 ^f^
	100	40.80 ± 5.45 ^e^	9.60 ± 2.08 ^e^	-	14.37 ± 2.68 ^e^
	500	130.40 ± 5.03 ^d^	15.20 ± 3.27 ^d^	-	55.63 ± 2.34 ^d^
	1000	490.20 ± 3.49 ^c^	27.60 ± 2.08 ^c^	-	242.09 ± 4.72 ^c^
	1500	595.20 ± 4.49 ^b^	47.20 ± 4.44 ^b^	-	372.93 ± 5.19 ^b^
	2000	870.60 ± 4.39 ^a^	71.60 ± 3.36 ^a^	-	674.91 ± 4.85 ^a^
14	Control	0 ± 0 ^f^	0 ± 0 ^f^	-	0 ± 0 ^f^
	100	55.20 ± 4.60 ^e^	20.40 ± 4.51 ^e^	-	15.53 ± 4.54 ^e^
	500	233.00 ± 3.39 ^d^	24.80 ± 4.15 ^d^	-	75.17 ± 3.30 ^d^
	1000	581.60 ± 5.03 ^c^	46.00 ± 3.16 ^c^	-	243.06 ± 4.37 ^c^
	1500	704.80 ± 4.32 ^b^	61.60 ± 3.51 ^b^	-	350.13 ± 3.77 ^b^
	2000	1307.40 ± 3.58 ^a^	92.20 ± 3.70 ^a^	-	680.96 ± 3.87 ^a^
21	Control	0 ± 0 ^f^	0 ± 0 ^f^	0 ± 0 ^f^	0 ± 0 ^f^
	100	63.40 ± 3.21 ^e^	29.80 ± 3.19 ^e^	11.40 ± 2.88 ^e^	15.97 ± 1.19 ^e^
	500	311.20 ± 4.32 ^d^	47.60 ± 3.64 ^d^	24.60 ± 3.21 ^d^	91.69 ± 5.35 ^d^
	1000	643.80 ± 4.92 ^c^	54.80 ± 2.59 ^c^	31.20 ± 3.35 ^c^	251.55 ± 5.34 ^c^
	1500	791.40 ± 3.85 ^b^	86.40 ± 4.62 ^b^	44.40 ± 3.71 ^b^	369.33 ± 4.61 ^b^
	2000	1376.60 ± 3.97 ^a^	104.80 ± 3.11 ^a^	50.80 ± 3.19 ^a^	672.97 ± 3.59 ^a^
28	Control	0 ± 0 ^f^	0 ± 0 ^f^	0 ± 0 ^f^	0 ± 0 ^f^
	100	80.20 ± 2.17 ^e^	30.60 ± 3.71 ^e^	27.20 ± 3.49 ^e^	18.88 ± 1.55 ^e^
	500	397.00 ± 2.92 ^d^	63.20 ± 3.35 ^d^	35.40 ± 2.88 ^d^	110.99 ± 2.86 ^d^
	1000	703.80 ± 4.32 ^c^	70.20 ± 4.55 ^c^	44.20 ± 3.35 ^c^	230.34 ± 3.31 ^c^
	1500	893.60 ± 4.22 ^b^	105.80 ± 4.98 ^b^	50.20 ± 4.55 ^b^	402.79 ± 3.22 ^b^
	2000	1576.60 ± 4.83 ^a^	117.40 ± 5.31 ^a^	63.6 ± 3.29 ^a^	704.86 ± 2.97 ^a^

Data represents Mean ± standard deviation (*n* = 5). The different letters inside a column represent the significance difference according to Duncan’s multiple range test (*p* < 0.05).

## Data Availability

The authors confirm that the data supporting the findings of this study are available within the article.
